# Acute Phase Neuronal Activity for the Prognosis of Stroke Recovery

**DOI:** 10.1155/2019/1971875

**Published:** 2019-09-08

**Authors:** Filippo Zappasodi, Patrizio Pasqualetti, Paolo M. Rossini, Franca Tecchio

**Affiliations:** ^1^Department of Neuroscience, Imaging and Clinical Sciences, “G. d'Annunzio” University of Chieti, Chieti 66100, Italy; ^2^Institute for Advanced Biomedical Technologies, “G. d'Annunzio” University, Chieti 66100, Italy; ^3^Medical Statistics and Information Technology, Fatebenefratelli Foundation for Health Research and Education, AFaR Division, Rome 00186, Italy; ^4^Institute of Neurology, Department of Geriatrics, Neurosciences & Orthopaedics, Catholic University of Sacred Heart, Rome 00168, Italy; ^5^Policlinic Gemelli Foundation, IRCCS, Rome 00168, Italy; ^6^Laboratory of Electrophysiology for Translational neuroScience (LET'S)-ISTC-CNR, Rome 00185, Italy

## Abstract

Strokes causing similar lesions and clinical states can be followed by diverse regains of neurological functions, indicating that the clinical recovery can depend on individual modulating factors. A promising line to disclose these factors, to finally open new therapeutic strategies, is to search for individual indices of recovery prognosis. Here, we pursued on strengthening the value of acute phase electrophysiological biomarkers for poststroke functional recovery in a wide group of patients. We enrolled 120 patients affected by a monohemispheric stroke within the middle cerebral artery territory (70 left and 50 right damages) and collected the NIH stroke scale (NIHSS) score in the acute phase (T0, median 4 days) and chronic follow-up (T1, median 6 months). At T0, we executed electrophysiological noninvasive assessment (19-channel electroencephalography (EEG) or 28 channels per side magnetoencephalography (MEG)) of brain activity at rest by means of band powers in the contra- and ipsilesional hemispheres (CLH, ILH) or the homologous area symmetry (HArS). Low-band (2-6 Hz) HArS entered the regression model for predicting the stabilized clinical state (*p* < 0.001), with bilateral impairment correlated with a poor outcome. Present data strengthen the fact that low-band impairment of homologous ipsi- and contralesional hemispheric regions in the acute stroke indicate a negative prognosis of clinical recovery.

## 1. Introduction

It is a common experience that after stroke the patients' clinical course is largely variable despite a nearly identical early clinical picture and similar size and location of the lesion [1]. In this scenario, we move on searching for individual features with prognostic value about the final outcome, which would help to better elucidate the mechanisms of poststroke functional recovery and to provide prospectively a guide in the selection of personalized rehabilitation treatments. Aware that stroke is a leading cause of disability [2], we searched for changeable factors indicating potential targets of sensorimotor rehabilitation enrichments. Previous studies revealed a clear neurovascular uncoupling in stroke patients [3] with preserved electrophysiological activity even in the presence of impaired hemodynamics [4]. Taking into account that neuronal plasticity definitely supports recovery abilities [5–8], and that it is mediated by changes of the neuronal electric activity, neuronal electric activity features per se are good candidates when searching for prognostic markers about clinical recovery. Furthermore, the existence of neuromodulation interventions enhancing recovery from stroke (for review, [9–12]) strengthens the relevance of electrophysiological prognostic markers to better tailor such interventions in compensating specific alteration in individual patients. On these bases, while it is crucial to operate the best of knowledge in limiting the lesion dimension by proper interventions in the first hours after the stroke [13], we will devote our investigation on the electrophysiological assessment of neuronal activity after patients' vital parameter stabilization, in the 2-10 days from the symptom onset.

Our aim was to strengthen the value of acute phase electrophysiological biomarkers for poststroke functional recovery in a wide group of patients suffering from a monolateral middle cerebral artery (MCA) stroke. We moved from the knowledge that the balances of EEG rhythm powers between interhemispheric homologous areas [14] and the low-band power of the contralesional hemisphere [15, 16] provide information about the clinical recovery ability after stroke. In diverse clinical conditions, the dynamic interplay between homologous cortical areas was a critical element for a proper functioning of the motor system either during task execution or even at rest. Notably, the behavioral performance associates with the functional connectivity across the nodes of the devoted networks in a resting state [17–19]. Thus, here, we focused on the interhemispheric balance at rest between the neuronal activities of areas supplied by the MCA. Deriving the neuronal activity from noninvasive electrophysiological recordings, i.e., electro- and magnetoencephalography (EEG and MEG), we obtained a normalized index of homologous area balance. We finally considered the hemispheric values to elucidate the local impairments accounting for the occurring imbalances.

## 2. Materials and Methods

### 2.1. Subjects

We enrolled 120 patients (mean age 70.6 ± 11.0 years, 75 men and 45 women) admitted to our departments (S. Giovanni Calibita Hospital and Fondazione Policlinico Agostino Gemelli, Rome) for a first-ever monohemispheric and monolesional ischemic stroke in the MCA territory. The inclusion criteria were clinical evidence of sensory-motor deficit of the upper limb and neuroradiological diagnosis of ischemic brain damage in MCA territory. The exclusion criteria were previous stroke on clinical history, neuroradiological evidence of involvement of both hemispheres or of brain hemorrhage, and dementia or aphasia severe enough to impair patients' compliance with the procedures. Patients received the best clinical care according to the Italian stroke guidelines (SPREAD).

Thirty-three healthy volunteers, matched for age and gender with patients, were also enrolled as the control group (mean age 70.0 ± 11.6 years, 20 males, 13 females, independent *t*-test for age between patients and controls: *p* = 0.770). All subjects of the control group were right-handed, as confirmed by the Edinburgh Manuality test, were not receiving any psychoactive pharmacological treatment at the time of recordings, and resulted normal at both neurological and brain magnetic resonance examinations.

The Ethics Committees of our hospitals approved the experimental protocol (Fatebenefratelli EC 40/2011), and all patients and healthy subjects signed a written informed consent before participating.

### 2.2. Data Collection

Clinical scores, EEG or MEG recordings, and MRI evaluation were collected in patients during the same day, after stabilization of the vital parameters and always before day 10 from the symptom onset (T0). Clinical scores were also collected in the postacute stabilized phase after 6 months (T1). The neurological assessment of stroke severity was executed by an accredited neurologist via the NIH stroke scale (NIHSS). The same neurologist scored the scale both at T0 and at T1. We decided to assess the clinical state by the NIHSS score even in the stabilized T1 phase to better serve the aim of our study. This choice was done to quantify the recovery processes separating from the stabilized phase clinical conditions the changes with respect to the acute phase state. Thus, we calculated the “effective recovery” (ER) as the percentage of the occurred improvement with respect to the total possible improvement, taking into account that NIHSS = 0 corresponds to the absence of clinical symptoms:

(1) $$\begin{array}{c} \text{ER}=100*\frac{\text{NIHSS at t}0-\text{NIHSS at t}1}{\text{NIHSS at t}0-0}. \end{array}$$

The brain MRI was carried out at 1.5 T Spin-Echo, Turbo Spin-Echo, using fluid-attenuated inversion recovery sequences. All sequences provided contiguous 5 mm thick slices on sagittal, coronal, and axial planes. The identification of the lesion site was performed on axial slices. Lesions were classified as “cortical” (C), if the cortical grey matter was involved and all subcortical structures were spared; as “subcortical” (S), when the white matter, internal capsule, thalamus, or basal ganglia were affected; and finally, as “cortico-subcortical” (CS), when both the cortical and the subcortical structures were involved.

A five-minute open-eye electroencephalographic (EEG) or magnetoencephalographic (MEG) recording was acquired at rest, while subjects sat on a comfortable armchair or lied on a hospital bed. Eighty patients (mean age 71.2 ± 9.8 years, 29 women) and 20 healthy controls (mean age 71.5 ± 6.4 years, 7 women, independent *t*-test for age between patients and controls: *p* = 0.895) underwent EEG recording, while 40 patients (mean age 69.4 ± 13.1 years, 16 women) and 13 healthy controls (mean age 67.7 ± 16.8 years, 6 women, independent *t*-test for age between patients and controls: *p* = 0.705) completed MEG examination.

The EEG activity was recorded by 19 Ag-AgCl cup electrodes positioned according to the 10–20 international EEG system (F1, F7, T3, T5, O1, F3, C3, P3, FZ, CZ, PZ, F2, F8, T4, T6, O2, F4, C4, and P4) in fronto-central reference; an additional electrode pair served for recording electrooculogram to control for eye blinking. Electrocardiogram was monitored by one bipolar channel placed on the chest. EEG data were sampled at 256 Hz (presampling analogical filter 0.1-70 Hz) and collected for offline processing. The MEG activity was recorded by a 28-channel system (16 inner axial gradiometers, 8 cm baseline and 9 mm pick-up coil diameter; 9 peripheral squared magnetometers, 9 mm pick-up coil edge; and three balancing magnetometers devoted to noise reduction) covering a scalp area of about 180 cm^2^, inside a magnetically shielded room (Vacuumschmelze GmbH). We recorded brain magnetic fields from the parietofrontal region of each hemisphere, by centering the sensor array on C3 and C4 of the international 10–20 electroencephalographic system. The system positioning was selected to assess cortical sensorimotor area activity, mostly affected by the lesion [20–23]. MEG data were sampled at 1000 Hz (presampling analogical filter 0.48-250 Hz) and collected for offline processing.

### 2.3. Data Analysis

**Figure 1:**
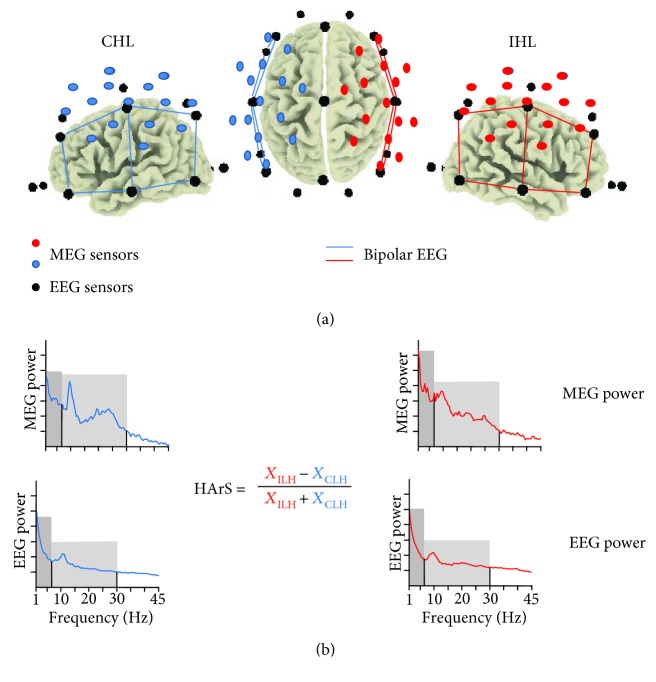
(a) International 10–20 system electrode positions in relation to the cerebral cortex (black circles). In a representative subject with the lesion in the right hemisphere, coloured bars show bipolar derivations overlying the MCA territory, used in our experiment to assess EEG spectral powers (red for the hemisphere ipsilateral to the lesion (ILH) and blue for the hemisphere contralateral to the lesion (CLH)). Red (ILH) and blue (CLH) circles indicate the positions of the 16 gradiometers in each hemisphere used to assess MEG spectral powers. (b) Spectral power densities were separately calculated in the ILH and CLH as the mean of those of bipolar derivations overlying MCA territory (EEG signals) or as the mean of those of gradiometers (MEG signals). Spectral power densities are shown in two exemplificative patients (MEG and EEG signals). We evidenced DeltaTheta (dark grey) and AlphaBeta (light grey) bands. The homologous area symmetry (HArS) index is calculated as shown for band and total powers.

A semiautomatic procedure based on Independent Component Analysis [24] was applied to both MEG and EEG data, in order to identify and eliminate artefacts (i.e., eye movements, cardiac activity, and scalp muscle contraction) without epoch exclusion. For EEG data, bipolar derivations between pairs of first-near electrodes in posterior-anterior and mediolateral directions were estimated selecting the sites overlying the MCA territory and maintaining separated the measures in the two hemispheres: F3-F7, C3-F3, F7-T3, C3-T3, C3-P3, T3-T5, and P3-T5 for the left hemisphere and the F4-F8, F4-C4, F8-T4, C4-T4, C4-P4, T4-T6, and P4-T6 for the right (Figure 1).

We estimated the Power Spectral Density (PSD) for each EEG derivation or MEG channel via the Welch procedure, using time windows of 4 s duration (resulting in a frequency resolution of 0.25 Hz), Hanning windowing, 60% overlap, and about 70 artefact-free trials. The PSD was calculated as the mean of the PSDs obtained for the 7 EEG bipolar derivation (EEG data) or by the 16 inner gradiometer channels (MEG data) separately in the hemisphere ipsilateral to the lesion (ILH) and the hemisphere contralateral to the lesion (CLH). The individual alpha frequency (IAF) peak was firstly calculated as the frequency with maximal PSD in the 7-13.5 Hz interval in parietooccipital regions. Then, as slow frequency has been linked to clinical status, lesion side, and recovery [25–28] and 10-20 Hz activity has physiological relevance in sensorimotor areas (mu rhythm [29, 30]), we considered the following frequency bands: DeltaTheta (from 2 to the minimum between 7.5 Hz and IAF-2 Hz) and AlphaBeta (from IAF-2 Hz to 30 Hz) according to previous stroke studies [31, 32].

The degree of symmetry of homologous MCA areas (HArS), i.e., between ILH and CLH activity, was obtained for the different bands and total power as [14, 33]:

(2) $$\begin{array}{c} \text{HArS}=\frac{X_{\text{IHL}}-X_{\text{CHL}}}{X_{\text{IHL}}+X_{\text{CHL}}}, \end{array}$$

being *X* the power in the DeltaTheta band, in the AlphaBeta band, or in the whole spectrum.

### 2.4. Statistical Analysis

Statistical analyses were performed using SPSS v. 16 statistical software (Chicago, Illinois, USA), and 0.05 was considered as the significance threshold. All values (band and global HArSs and hemispheric powers) were log transformed to better fit a normal distribution for statistical analysis (checked by the Shapiro-Wilk test) when needed. Moreover, they were controlled not to differ between MEG and EEG groups.

The statistical analysis is aimed at testing whether the interhemispheric activity unbalance, measured by the HArS index, provides prognostic information about the clinical recovery from stroke in the stabilized phase, as measured by NIHSSs. We preliminarily selected the HArS variables which add a prognostic information with respect to the clinical state in the acute phase, applying a regression model with NIHSS at T1 as a dependent variable and NIHSS at T0, total and band HArS values as independent variables. After this selection, we better depicted the link between HArS and the clinical state in the stabilized phase (NIHSS at T1) or effective recovery (ER) by means of Spearman's or Pearson's correlation.

To clarify the phenomena behind the interhemispheric unbalances related to clinical recovery with possible dependence on the lesion side, we applied ANOVA for repeated measures on corresponding band powers with *Hemisphere* (left, right) as the within-subjects factor and *Group* (left lesion, right lesion, and healthy control) as the between-subjects factor. Whenever the interaction *Hemisphere*∗*Group* effect was found, the significance of the post hoc comparisons between the groups for each hemisphere was assessed-corrected by Bonferroni's procedure.

For the correlative analysis, to develop a measure independent of the laboratory, we derived *z* scores for band and total powers. Specifically, for each hemisphere and separately for MEG and EEG groups, we divided patients' values for the standard deviation of the distribution of healthy controls, after subtracting the mean of the values of healthy controls. We note that in this way the measure is even independent of EEG/MEG investigation, although band and total powers differ depending on the MEG or EEG assessment. To assess the robustness of the results, a percentile-based bootstrap, with 5000 replicate samples, was performed to assess the 95% confidence intervals of correlation coefficients.

## 3. Results and Discussion

### 3.1. Patients' Picture

The NIHSS score in the acute phase (T0) was collected at a median of 4 days (between 1 and 10 days) after the stroke onset. NIHSS at T0 ranged from 1 to 22 (median: 5.0; 5-95 percentile: 1-18). As assessed by NIHSS at T1 with respect to T0, all patients showed at least some clinical recovery, with the exception of 3 patients with a cortico-subcortical lesion in the right hemisphere who showed a worsened clinical picture at T1 and 5 patients who did not change clinical status at the two times. Thirty-four patients showed a complete recovery (ER = 1). Right-lesion and left-lesion patients did not differ for NIHSS in the acute phase, for NIHSS in the stabilized phase, for recovery, or for age (Table 1). Moreover, the clinical picture was not different between patients who underwent EEG or MEG (Table 1). According to the ischemic injury localization, 13 patients (11%) were classified as cortical, 39 (33%) as subcortical, and 68 (56%) as cortical-subcortical.

The following risk factor percentage was present in the recruited stroke population: 21% smoking, 23% diabetes, 69% hypertension, 29% cardiopathy (13% atrial fibrillation), 65% hyperlipidaemia, and 10% familiarity.

### 3.2. Prognostic Analysis: Homologous Area Symmetry (HArS)

HArS variability was only marginally accounted for by technique MEG/EEG groups (eta − square = 0.006, *p* = 0.413); therefore, HArSs were studied in the whole group of the 120 patients.

The regression analysis with NIHSS at T1 as a dependent variable included the clinical status in the acute phase and total and band HArS as independent variables. In addition to NIHSS at T0, HArS in DeltaTheta entered the model, as expressed by

(3) $$\begin{array}{c} \left(\text{NIHSS at T}1\right)=-2.0+0.75\;\left(\text{NIHSS at T}0\right)-25.73\;\left(\text{DeltaTheta HArS}\right). \end{array}$$

The 73% of the variance of NIHSS at T1 was explained by this model (*F*(2,117) = 152.608, *p* < 0.001). The signs of the coefficients tell us that, as expected, a worse clinical status at T0 correlates with a worse clinical status at T1. Furthermore, a smaller DeltaTheta interhemispheric symmetry in the acute phase correlates with a better clinical picture in the stabilized phase.

### 3.3. Hemispheric ILH and CLH Powers

**Figure 2:**
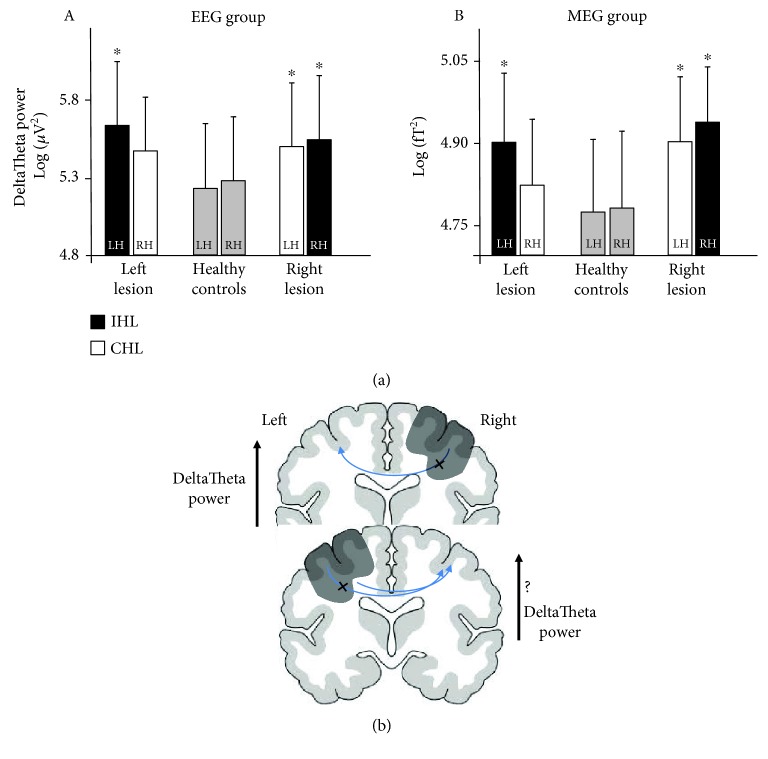
Asymmetric impact of a left or right lesion. (a) For the EEG group (A) and the MEG group (B): mean (standard deviation) of DeltaTheta band powers of left and right hemispheres in patients with the lesion in the left hemisphere, healthy controls, and patients with the lesion in the right hemisphere. For patients, the black bar indicates the hemisphere ipsilateral to the lesion (ILH) and the white bar indicates the hemisphere contralateral to the lesion (CLH). Asterisk indicates that the post hoc independent *t*-test with respect to the value of the corresponding hemisphere in healthy controls is significant (Bonferroni-corrected). (b) A schematic representation of the functional asymmetry of the right/left interhemispheric projections (see Results and Discussion).

To understand the origin of higher DeltaTheta asymmetries correlated with better recovery levels, we analysed the subtending hemispheric powers. To discriminate phenomena possibly depending on right vs. left lesions, we executed a repeated measures ANOVA design on DeltaTheta band power with *Hemisphere* (left, right) as a within-subjects factor and *Lesion Side* (lesion in the left hemisphere, lesion in the right hemisphere, no lesion = healthy control) as a between-subjects factor. A clear interaction *Hemisphere*∗*Lesion Side* was found (*F*(1, 78) = 10,901; *p* = 0.001, EEG group; *F*(1, 38) = 7,160; *p* = 0.011, MEG group). Post hoc comparisons with respect to controls (Bonferroni-corrected, Figure 2) showed that left-damaged patients had DeltaTheta power increased in the ILH, and right-damaged patients had a bilateral increase (ILH and CLH, Figure 2). We can observe correspondingly that the DeltaTheta symmetry is higher in the right-damaged patient than left-damaged patients (DeltaTheta HArS values: 0.004 ± 0.019 vs. 0.012 ± 0.023, respectively; independent *t*-test: *t*(118) = 2.136; *p* = 0.035). Notably, after *z*-transformation (see Materials and Methods), we selected those patients with DeltaTheta CHL power higher than the 97,7% of DeltaTheta of controls (*z* score = ±2). They were 18 (15% of the 120 patients) and displayed a higher symmetry with respect to the other 102 patients (DeltaTheta HArS: 0.004 ± 0.020 vs. 0.011 ± 0.020, independent sample *t*-test: *t*(116) = 2.704; *p* = 0.008). Consistently, they showed a lower clinical recovery at T1 (ER: 40% ± 36% vs. 70% ± 31%, independent *t*-test: *t*(116) = 3.530; *p* = 0.001). Furthermore, higher CHL DeltaTheta correlated with worse clinical recovery (Table 2). We note that the same relationships on the predictive value of contralesional low-band activity hold for randomly chosen independent groups in the enrolled sample (Table 2).

## 4. Discussion

The main result of our study is that following a monohemispheric stroke in the middle cerebral artery territory, the bilateral increase of the brain low-band activity expressed in the increase of interhemispheric symmetry of the homologous areas' powers in the acute phase predicts a worse functional outcome in the stabilized phase.

We posed the working hypothesis that the homologous area activity balance was “the best” prognostic indicator, consistent with the clear achievement that the functional interhemispheric balance serves the network functionality. Interhemispheric unbalance has been recently observed in diverse neurological diseases [34–38], and the evidence of its functional role originated from the results of several studies in animal models and humans, in the consequence of an acquired brain lesion. In animal models, a parallel trend emerged between interhemispheric connectivity and neurological improvement after cerebral ischemia, longitudinally followed up from acute to chronic stages [39]. In humans, both fMRI and electrophysiological data in acute and chronic stroke patients demonstrated that the balance of these hemispheric areas associates with a better clinical picture [18, 33, 39–41]. Moreover, a functional interhemispheric uncoupling in the acute phase can lead to adverse prognostic consequences [16], and the interhemispheric asymmetry of complexity of the EEG dynamics in the acute phase is paired to a worse clinical status [42]. In this framework, our data express that the interhemispheric symmetry in the acute phase predicts a worse outcome as an expression of the increased contralesional hemispheric low-frequency activity.

We had considered the interhemispheric symmetry index between homologous areas as a good indicator because it is a parameter largely independent of the recording technique and settings. In this direction, we can consider a strength more than a weakness to include both EEG and MEG data, providing a clear consistency of the results independent of the assessing technique. Our work indicated that also hemispheric powers are informative, in particular via *z* scores which also minimize dependence on specific recoding settings. *z* scores depend on the quality of the normative population, which can be ameliorated by increasing the samples in the future.

Damaged areas typically generate delta rhythms [26, 28, 43]. This perilesional low-frequency activity is positively correlated with a worse clinical status in the acute phase [15] but does not add prognostic information with respect to the clinical severity. From a prognostic perspective, the most stable achievement from literature is a negative indication associated with a power increase of the low-frequency range of oscillatory neuronal activity from the CLH [31]. Here, we confirmed our findings of the prognostic value of CHL low-frequency activity found in studies involving independent cohorts [15, 16, 44] or by other authors who considered hemispheric phenomena [45, 46]. We documented that when CLH neuronal low-band activity emerges in addition to the typical increase in ILH one, this phenomenon adds prognostic power to the clinical severity in the acute phase indicating a poor functional outcome. Acute phase increase of delta power in CLH is secondary to transcallosal diaschisis, a more or less transient alteration of brain function remote from the lesion, according to von Monakow, who coined the term in a pre-EEG epoch [47]. Signs that the CLH power increase was mediated by an impairment of local contralesion inhibitory networks secondary to a loss of modulatory projections from damaged areas can be traced from the behavior after a right or left damage. In fact, we found that a right lesion typically induces a bilateral power increase, while a left one does it more rarely. Conceivably, this is consistent with the stronger inhibitory projection from the left onto the right hemisphere, which results more resistant to the damage. Conversely, a weaker inhibitory projection of the right onto the dominant left hemisphere corresponds to a right damage impacting more significantly the left dominant region (Figure 2(b)). Accordingly, functional evidence indicates that in rightward subjects, interhemispheric inhibition phenomena are asymmetric [48], with the left sensorimotor regions inhibiting the right more than the other way around (transcranial magnetic stimulation (TMS) studies [49, 50]; functional magnetic resonance imaging (fMRI) studies [51]).

Different from the role on the lesioned hemisphere, we did not investigate deeply the electrophysiological alterations independent on the lesion localization and extension. This is in agreement with the study design depending on the working hypothesis: when searching for individual features revealing prognosis of recovery, we typically expect factors that are independent on the lesion site and dimension. In this respect, we moved according to our focus on tracking measures in the acute phase associated with diverse regains of neurological functions despite similar lesions and clinical states.

Our main perspective scope is to find prognostic measures about recovery ability to use as biomarkers for patient selection in designing rehabilitation treatments and/or noninvasive neuromodulation protocols.

Our study has a number of limitations. First, all our EEG estimates were derived by homologous bipolar derivations. In the future, it can be that by better evaluating homologous brain neuronal pools via measures derived on the cerebral sources' activities, we will strengthen the relationships with recovery. Supportive of this idea, in chronic stroke patients, we measured the connectivity between lesional and contralesional sensorimotor regions, by either considering the bipolar-EEG activity as in the present investigation or focusing on the cerebral sources in sensorimotor regions devoted to the paretic and the nonparetic hand [36]. Exclusively, the assessment via the source activities revealed the association with robot-aided rehabilitation effects [40]. Furthermore, we evaluated the clinical state through NIHSS, a suitable scoring in the acute state but roughly assessing the finer functionality of the patient in the stabilized condition. Since the present investigation focuses on acute phase markers correlated with the improvement of the clinical state, we preferred to obtain a relative index of the clinical improvement reached by the patient (normalized by the total possible improvement), instead of using scales proper to assess the patient's functional abilities and everyday independence in T1 (Modified Rankin Scale, Bartlett Index, and Fugl-Meyer) which are not collected in the acute phase; thus, they do not allow a differential T1 vs. T0 evaluation.

## 5. Conclusions

The interhemispheric homologous areas' low-band power symmetry predicted the functional recovery ability in addition to the clinical state at symptoms' onset, reflecting a power increase of the contralesional hemisphere. A more frequent bilateral increase occurred after a right than left damage. The present data strengthen the notion that proper neuromodulations in acute stroke can enhance recovery abilities and provide suggestions on how to personalize the intervention (select people depending on the HArS value, apply bilateral inhibitory NIBS).

## Figures and Tables

**Table 1:** Demographic, clinical, and neuroradiological picture of the 120 monohemispheric MCA stroke patients.

	EEG (*n* = 80)						MEG (*n* = 40)						*Comparison*	
Lesion side	Left			Right			Left			Right			*EEG vs. MEG*	*L vs. R lesion*
Number *(% of EEG/MEG group)*	46 *(58%)*			34 *(42%)*			24 *(60%)*			16 *(40%)*				
Gender	Male		Female	Male	Female		Male	Female		Male	Female		*0.694*	*0.849*
*Number (% of lesion side group)*	28 *(61%)*		18 *(39%)*	23 *(68%)*	11 *(32%)*		15 *(64%)*	9 *(36%)*		9 *(56%)*	7 *(44%)*			
Age (*mean* ± *st* *dev*)	70.0 ± 8.6			72.8 ± 11.2			68.8 ± 12.2			70.3 ± 14.8			*0.389*	*0.239*
Lesion class	*C*	*S*	*CS*	*C*	*S*	*CS*	*C*	*S*	*CS*	*C*	*S*	*CS*	*0.451*	*0.404*
	5	19	22	3	11	20	3	6	15	2	3	11		

Clinical picture														
NIHSS at T0 *Median (5-95 perc.)*	**6** (1–20)			**5** (1–18)			**5** (2–14)			**5** (2–14)			*0.565*	*0.307*
NIHSS at T1 *Median (5-95 perc.)*	**2** (0–18)			**2** (0–11)			**1** (0–8)			**3** (0–10)			*0.474*	*0.785*
Effective recovery *Median (5-95 perc.)*	**70** (9–100)			**73** (30–100)			**83** (1–100)			**67** (-22–100)			*0.500*	*0.855*

In the last 2 columns, *p* values of the statistical test are shown, which were used to compare EEG vs. MEG groups and patients with the lesion in the left or in the right hemisphere; independent sample *t*-test (age, ER), chi-square (gender, lesion class), and Mann-Whitney test (NIHSS in acute and stabilized phases).

**Table 2:** Correlations between spectral band powers and clinical variables.

	DeltaTheta		AlphaBeta		Whole band	
	ILH	CLH	ILH	CLH	ILH	ILH
NIHSS at T0	0.416 (0.251, 0.560) <0.001	0.312 (0.134, 0.469) 0.001	n.s.	n.s.	0.181 (-0.010, 0.360) 0.048	0.208 (0.025, 0.376) 0.023

NIHSS at T1	n.s.	**0.285** (0.119, 0.432) 0.002	n.s.	n.s.	n.s.	n.s.

ER	n.s.	**-0.289** (-0.123, -0.431) 0.002	n.s.	n.s.	n.s.	n.s.

Correlation coefficients (confidence limit in the second line, assessed by the bootstrap procedure, and *p* value in the third line) of *z*-scored band and total powers in ipsilesional (ILH) and contralesional (CLH) hemispheres with an acute clinical score (NIHSS at T0 and at T1—Spearman rho), clinical score in the stabilized phase (^∗^NIHSS at T1 adjusted for NIHSS at T0), and effective recovery (ER—Pearson *r*) both adjusted for NIHSS at T0 (^∗^Pearson *r*). Values in bold are for significance < 0.050.

## Data Availability

EEG raw data, personal and clinical anonymized data will be available upon reasonable request.
